# Revisiting fast-charging performance in supercapacitors

**DOI:** 10.1093/nsr/nwag164

**Published:** 2026-03-14

**Authors:** Changde Ma, Feng Zhou, Zhong-Shuai Wu

**Affiliations:** State Key Laboratory of Catalysis, Dalian Institute of Chemical Physics, Chinese Academy of Sciences, China; State Key Laboratory of Catalysis, Dalian Institute of Chemical Physics, Chinese Academy of Sciences, China; State Key Laboratory of Catalysis, Dalian Institute of Chemical Physics, Chinese Academy of Sciences, China; Dalian National Laboratory for Clean Energy, Chinese Academy of Sciences, China

Electrochemical double-layer capacitors (EDLCs), also known as supercapacitors, are valued for their rapid charge–discharge capability and long cycling life [1,2], rendering them well suited for applications that demand high power density. These include hybrid and electric vehicles, power grid frequency regulation, electromagnetic launch systems and portable electronic devices. Nevertheless, efforts to enhance their energy density have often overshadowed rate capability, a performance metric critically governed by electrode materials and electrolyte properties [3,4]. Despite the presumed importance of mesoporosity for ion transport, the relationship between pore architecture, ion dynamics and electrochemical performance remains poorly understood. This knowledge gap stems largely from the limitations of conventional characterization techniques in probing ion transport within complex porous networks, particularly in commercial nanoporous carbons. Consequently, there is an urgent need for experimental strategies capable of directly interrogating electrolyte transport within pore networks to identify the key factors governing ion transport and rate capability.

**Figure 1.**
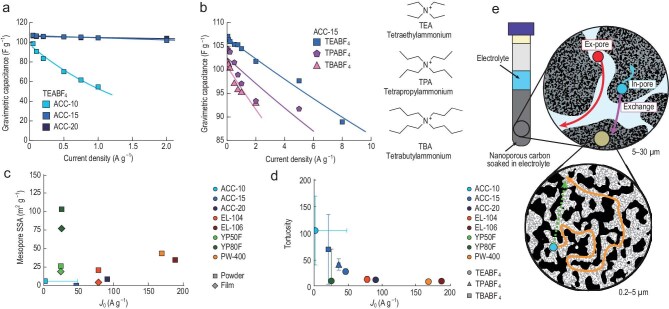
(a) Electrochemical rate performance of ACC electrodes with varying pore size distributions in 1 M TEABF_4_/ACN. ACN, acetonitrile. (b) Rate capability of ACC-15 in electrolytes containing cations of increasing size (TEA^+^, TPA^+^ and TBA^+^) dissolved in ACN. (c) Correlation between rate capability and SSA of mesopores from gas sorption. (d) Correlation between pore-network tortuosity and rate capability. (e) Schematic of diffusion in nanoporous carbons. Adapted with permission from Kress *et al*. [5].

In a recent study, Kress *et al.* employed diffusion pulsed-field gradient (PFG) nuclear magnetic resonance (NMR) to directly quantify the effective anion diffusion coefficients within nanoporous carbon electrodes, with a particular focus on elucidating the relationship between ion transport in nanoporous carbons and the fast-charging performance of EDLCs [5]. Electrochemical characterization of a series of activated carbon cloths (ACCs) revealed comparable specific capacitances at low current densities, yet marked differences in rate performance emerged at higher rates (Fig. 1a). Further investigation using a fixed electrode material (ACC-15) with electrolytes of varying cation sizes showed that larger cations systematically degrade fast-charging capability (Fig. 1b).

A systematic analysis of mesoporosity indicates that the mesopore fraction (2–50 nm), despite being widely regarded as a critical parameter, is not intrinsically correlated with the rate capability of supercapacitors (Fig. 1c). By explicitly distinguishing short-range (<0.2 μm) from long-range (>3 μm) diffusion regimes for the first time, the study demonstrates that the short-range diffusion coefficient (*D*₀) is unrelated to rate performance, whereas the long-range diffusion coefficient (*D*_∞_) shows a strong correlation. By defining the tortuosity *τ* = *D*₀/*D*_∞_, the authors further establish that nanoporous carbons with lower tortuosity exhibit superior rate capability, identifying pore-network tortuosity as a key parameter controlling the charging kinetics of nanoporous carbons (Fig. 1d). Figure 1e schematically illustrates diffusion in nanoporous carbons, clearly distinguishing species outside the pores (red), anions exchanging between the pore interior and exterior (magenta) and a slowly diffusing component (blue). The contrast between the orange convoluted pathways and the green dashed straight lines visually highlights the tortuous nature of ion diffusion paths within the pore network.

This work further clarifies a long-standing misconception by demonstrating that the mesoporosity is not the determining factor for rate capability, and that the mesopore specific surface area (SSA) shows no evident correlation with the long-range diffusion coefficient. In addition, the study reveals that the size of electrolyte cations can indirectly regulate anion mobility by modulating pore accessibility. Importantly, PFG NMR is shown to be a powerful technique for resolving ion diffusion across multiple length scales, providing a robust and reliable tool for probing pore-network structure and transport properties. Moreover, pore connectivity has been shown to improve the rate capability of battery materials, and this approach holds promise for providing experimental insights into the mechanisms underlying high-rate performance.

In summary, by integrating PFG NMR with electrochemical measurements, Kress *et al.* elucidate the fundamental factors governing fast-charging performance in nanoporous carbon-based supercapacitors. Their findings establish that pore-network tortuosity and connectivity, rather than porosity alone, dictate ion transport kinetics and rate capability. This work advances a design paradigm shift: future electrode engineering should prioritize low-tortuosity, well-connected pore architectures to mitigate transport limitations, thereby enabling simultaneous enhancement of energy and power density. Beyond supercapacitors, these insights may also inform pore-structure engineering in other electrochemical energy storage systems, such as batteries and hybrid capacitors, where efficient ion transport within porous electrodes is equally critical to high-rate performance.
